# A grounded theory of cognitive analytic reflective practice groups

**DOI:** 10.1111/papt.12557

**Published:** 2024-11-11

**Authors:** Sasha Priddy, Stephen Kellett, Shona Goodall, Rachael Cotton

**Affiliations:** ^1^ Rotherham, Doncaster and South Humber NHS Foundation Trust Rotherham UK; ^2^ Clinical and Applied Psychology Unit University of Sheffield Sheffield UK; ^3^ Sheffield Children's NHS Foundation Trust Sheffield UK

**Keywords:** adolescence, cognitive analytic therapy, reflective practice

## Abstract

**Objectives:**

Whilst cognitive analytic therapy (CAT) is increasingly used as an indirect intervention, there is little evidence of how the approach can be applied to reflective practice. This study sought to develop a process model of cognitive analytic reflective practice (CARP) groups.

**Design:**

Constructivist grounded theory approach supplemented by quantitative measures of helpfulness and group cohesion.

**Methods:**

Twenty‐four participants, working within four staff teams in a secure children's home, attended four CARP groups over a 1‐year period. Sessions were audio recorded, transcribed and checked for model fidelity and then analysed using grounded theory. Theoretical sampling was achieved via conducting focus groups with teams informed by the emergent categories, and using sessional measures of group cohesion and helpfulness to confirm theoretical saturation.

**Results:**

The model constructed had three interrelated main categories: ‘facilitator processes: establishing a reflective space’, ‘group processes: widening awareness of the self, others, and system’ and ‘outcomes: changing relational dynamics and finding exits.’ The model was validated by evidence from the measures.

**Conclusions:**

The model offers an empirical understanding of how CAT informs reflective practice through a better understanding of reciprocity in the dynamics of care systems.

## INTRODUCTION

The Children and Young People's Secure Estate (CYPSE) is tasked with providing care and secure accommodation for young people (YP) in the United Kingdom, who are identified by the local authority as raising welfare concerns and/or are involved in the youth justice system. YPs within CYPSE are described as ‘high risk, high harm, and high vulnerability’, due to past trauma and current relational disruption and socioeconomic adversity (Taylor et al., 2018). The implementation of the ‘Secure Stairs’ framework was mandated to better meet the needs of YP through establishing a trauma and relationallyinformed, formulation‐driven and a whole‐systems approach (National Health Service England, 2018; Taylor et al., 2018). Whist it has been recommended that staff have access to regular reflective practice (RP) to help manage the complex relational demands and tasks of enabling and delivering care in these settings (Taylor et al., 2018), no single theoretical model has been championed.

This study concerns the delivery of cognitive analytic reflective practice (CARP) groups. Cognitive analytic therapy (CAT) is a brief, time‐limited, relational and integrative psychotherapy (Ryle & Kerr, 2020) that is now practiced internationally (Ryle et al., 2014). When used organisationally, CAT can formulate the complex interplay of service user, staff and wider organisational factors active in any care system (Kellett et al., 2014; Ryle & Kerr, 2002; Shannon et al., 2017). This recognises that components of the system can contribute equally via reciprocation, potentially creating and maintaining the service user's distress/dysfunction through ‘unconsciously replaying some of the chaos and complexity that the young people may have experienced in their lives’ (Taylor et al., 2018, p.194). When CAT has been used indirectly with care systems, this has been shown to improve team cohesion in a pilot randomised control trial (Kellett et al., 2014), drives formulation‐informed decision‐making (Stratton & Tan, 2019) and reduces staff burnout (Kellett et al., 2020).

CAT integrates aspects of theory and practice from personal construct (Kelly, 1955) and object relations theory (Ogden, 1983). The analytic component of CAT is *reciprocal roles* which summarise the ways in which developmental influences are internalised and then go onto influence self‐self, self‐other and other‐self relating (Ryle & Kerr, 2020). For the YP in care, reciprocal roles would summarise the way in which the YP relates to themself, the way in which the YP relates to the care system and the way in which the care system is invited to relate to the YP. Reciprocal roles would therefore be discussed during CARP. The cognitive components of CAT are conceptualised as *snags, traps and dilemmas* which describe the present‐day patterns maintaining distress or relational difficulties. This emphasis on relational dynamics is what makes CAT distinct from other psychological models (Ryle & Low, 1993). Analysis of relational patterns of care would therefore be a part of CARP.

A CAT‐specific model of RP (the ‘4Ps model’; Annesley & Jones, 2016) has been developed to better scaffold teams in being able to reflect on, conceptualise and have better insight into patient and staff team interactions/responses (Freshwater & Kerr, 2006). The 4Ps are ‘pause, pulls, patterns and professional reactions.’ The model aims to enable consistent empathy for service users, the creation and maintenance of consistent therapeutic behaviours, reduce toxic iatrogenic harm and improve boundary maintenance (Jones & Annesley, 2019). This would be an example of intelligent kindness (Campling, 2015). CAT has been adopted as a model in forensic services to structure and facilitate reflective practice groups (Marshall & Kirkland, 2021), and this also usefully differentiates CAT‐informed RP from generic RP. The distinguishing features of CAT‐informed RP is (a) that there is an assumption that the enactments with the service user are examples of the relational dynamics of the service user's history, (b) the use of reciprocal roles to understand enactments, (c) the capturing of parallel processes within the RP group and the relationship between the service user and the team, (d) active CAT mapping of roles and procedures taking place and (e) that exits to relational patterns can be created by the RP group.

There are also examples of using CAT directly with adolescents (Jenaway & Mortlock, 2008) and there is a well conducted randomised control trial of CAT with adolescents with emergent personality difficulties (Chanen et al., 2008). CAT was therefore well matched to the ‘Secure Stairs’ brief of providing a relationally‐informed approach to enable and support staff to be consistently therapeutic and so reduce the risk of iatrogenic interactions with the children and YP admitted to secure accommodation. CARP groups therefore had the brief of (a) supporting care staff teams to better understand reoccurring interpersonal challenges in the context of YP residents lived experiences and the reciprocal roles and associated procedures therefore being enacted, and (b) supporting teams to develop ‘exits’ out of patterns of behaviour, better navigation of reoccurring relational challenges and building confidence in consistency of care (Shannon et al., 2016). This research sought to explore how CARP groups influenced the dynamics of care in a secure children's home (SCH) by building a substantive grounded theory.

## METHOD

### Ethics, design and participants

This research was ethically approved (ref: 032422). Grounded theory (GT) was deemed an appropriate research methodology, as this study aimed to generate theory with explanatory power in a context where process was integral and there was limited extant research (Birks & Mills, 2011). Participants were staff members (*N* = 24) who had participated in (or facilitated) CARP within a SCH over 19‐month period across four staff teams. Participants included residential support workers (RSWs; *N* = 19), team leaders (TLs; *N* = 4) and a Clinical Psychologist (*N* = 1). The facilitator was a qualified Clinical Psychologist and ACAT accredited practitioner. See Table 1 for participant demographic information.

**TABLE 1:** Participant demographics.

Role	Gender		Age (years)		Previous exposure to CAT (prior to CARP attendance)		Years of experience in SCH context (or similar services)
	Male	Female	Mean	Range	Yes	No	Mean
RSW	9	10	35.62 (*SD* = 7.76)	24–48	1	23	5 (*SD* = 5.57)
Team lead	2	2	30.25 (*SD* = 2.5)	29–34	0	4	6.5 (*SD* = 1)

### RP facilitation and fidelity

All participants attended a 1‐day CAT workshop before the CARP groups started. Each team had four 60‐minute face‐to‐face CARP groups per year (one session facilitated remotely due to COVID‐19). The facilitator used an open agenda and CAT theory to structure the reflective discussions. An adapted version of the CAT competency measure (CCAT; Bennett & Parry, 2004) assessed the degree to which CAT was adhered to in two randomly selected sessions. The adapted version had 10 domains and was based on Kellett and Bennett's (2017) approach to using the CCAT to inform clinical supervision. The CCAT measures the psychotherapeutic competencies of CAT, and is achieved by listening to and rating sessions with a scoring manual and the scale. The CCAT scores competence across 10 domains (containing 77 elements), nine of which are generic, and one is CAT specific. The CCAT has a range of 0–40 and a cut‐off score of ≥20 being competent CAT. In the present study, CARP sessions were scored on whether each CAT criterion was ‘present/observed’ or ‘not observed’ by the lead researcher (SP) on the following items: phase‐specific therapeutic tasks, theory‐practice links, CAT‐specific tools and techniques, external framework, common factors, respect/commonality/mutuality, assimilation of warded of emotions/problematic states, making links/hypotheses, identifying/managing threats to the therapeutic alliance, and awareness/management of own reactions. Supporting Information provides a detailed breakdown of the CARP competencies, how these were defined and the session ratings. Percentages were calculated based on the number of domains present/observed across all domains; session one demonstrated 72% adherence and session two 57% adherence. Only two sessions were selected for CAT adherence ratings due to time constraints.

An NVIVO word frequency analysis was also conducted on all the CARP transcripts, to search for key CAT terminology and stemmed words. The search terms were CAT, reciprocal role, relational, enactment, pattern, sequence, attachment, state, state‐switch, map, zone of proximal development, boundaries, controller, pacifier, negotiator, transference, countertransference, reformulation, recognition, revision and exits. The number of references to these words within each CARP session and the overall mean references to CAT terminology were CARP1, 11; CARP2, 9; CARP3, 43; CARP4, 26; CARP5, 26; CARP6, 19; CARP7, 25; and CARP8, 16. The mean number of CAT specific utterances in the CARP groups was 21.88 (*SD* = 10.80).

### Data collection

**FIGURE 1:**
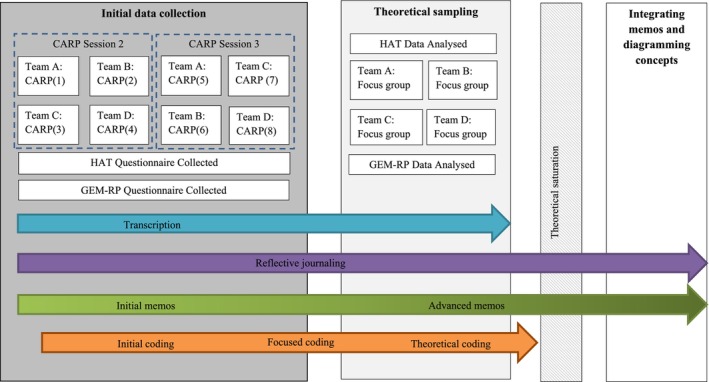
The GT development process based on the Charmaz (2006) framework.

The first stage of data collection used retrospective, purposeful sampling of recorded CARP group sessions. These were transcribed and provided data for the grounded theory. To aid saturation and support the iterative development of theoretical constructs, the second stage involved theoretical sampling, via a range of methods and measures. Data collection stages are illustrated in Figure 1. Based on the initial constructed categories from the analysis of the CARP sessions, a semi‐structured interview schedule was developed and 45–60‐minute focus groups conducted. Supporting Information contains the interview schedule that was used in the study. Consistent with GT principles, the interview schedule was modified considering any emerging data (Charmaz, 2006). To contextualise the data analysis, anonymised demographic data were collected in relation to relevant characteristics of the group members.

### Measures

The rationale for the measures used in the study were as follows; (a) that interviewing participants after each group would have been impractical and (b) the research wanted to access potential variation between CARP groups and variations over time. Whilst the Helpful Aspects of Therapy (HAT) measure has previously been used in therapy situations, it was easily adapted by simply changing the focus of the prompts. The Group Entitativity Measure–Group Psychotherapy measure is adaptable to any group situation, and as CARP is held within a group format, this was also deemed appropriate.

#### Helpful Aspects of reflective practice

An adapted version of the Helpful Aspects of Therapy Questionnaire (HAT; Llewelyn, 1988) collected data regarding helpful and/or hindering aspects of CARP groups. The measure had four questions, exploring ‘helpful’ and/or ‘unhelpful’ events within the CARP group session (e.g., ‘please describe what made this event helpful/important and what you got out of it’) and these were rated using a 9‐point Likert scale (i.e., 9 ‘extremely helpful’, 5 ‘neutral’, 1 ‘extremely hindering’).

#### Group Entitativity Measure–Group reflective practice

The Group Entitativity Measure–Group Psychotherapy (GEM‐GP; Hornsey et al., 2012) measured CARP group cohesiveness. Participants identified from six schematic drawings which image best represents how they perceived their team after each CARP group session.

### Data transcribing and analysis

All focus groups were transcribed using a naturalist word‐for‐word transcribing process to ensure data immersion (Oliver et al., 2005). Data coding involved three stages: initial, focused, and theoretical coding. Constant comparison was used to compare data with data, compare new data with emerging categories, and establish relationships between categories (Charmaz, 2006). Data sampling and analysis was iterative and continued until theoretical saturation was achieved (Birks & Mills, 2011).

### Coding

Initial coding was conducted using an incident‐by‐incident approach. This involved dividing each page of transcript into units of approximately 3–5 lines of text, collectively treated as an incident (Charmaz, 2006; Charmaz & Mitchell, 2001). Focused codes were identified through reviewing codes occurring frequently or that appeared particularly significant in communicating meaning (Charmaz, 2006). Categories were labelled using gerunds wherever possible, to ‘nudge’ the researcher towards active processes (Charmaz, 2006). Focused codes informed further data analysis and were revised in light of this data, resulting in constant refinement of codes. Through constant comparative analysis and memos, focused codes were then synthesised into tentative conceptual categories and subcategories (Charmaz, 2014). Theoretical coding enabled an integrated theory to be developed, with associated embedded interpretations regarding process.

### Quality assurance

The research adopted a social constructionist perspective (Burr, 2003), acknowledging that the researcher forms part of the meaning emerging from the data. A constructionist perspective was deemed most suitable, as it permits exploration of how relational interactions during CARP created versions of shared knowledge, rather than searching for a single objective truth within the data (Burr, 2003). The lead researcher also delivered a presentation of the proposed methodology to key SCH stakeholders involved in the commissioning of CARP and associated conversations were documented within the reflective log, to inform the research development process. During the development of the research design, CAT practitioners were consulted to ensure sensitivity to CAT practice in RP groups. Memo writing, using word documents and hand‐drawn diagrams or notes, was used throughout to explore developing ideas, and facilitate the formation of tentative hypotheses about patterns and processes occurring in the data (Charmaz, 2006). An example of a memo entries and associated transcript extracts are provided in Supporting Information. During the process of theoretical coding and analytic abstraction, CAT terms are used to aid with applicability and conceptual relevance. Research supervision and use of a reflective diary was used to counter potential bias. An example of reflective diary entry is provided in Supporting Information. Sensitivity to the participant's context was cross‐checked via reviewing ethnographic memos of the SCH environment. To assess whether emerging categories coherently explained the data, emerging categories were discussed with a peer researcher (Elliot et al., 1999). Member‐checking was used as part of the focus group process, to explore alternative constructions and researcher biases. To demonstrate the procedural logic used to interpret the data, quotes have been included throughout reporting of results. An audit trail to log memos against the development of categories was also used.

## RESULTS

### Qualitative findings

**FIGURE 2:**
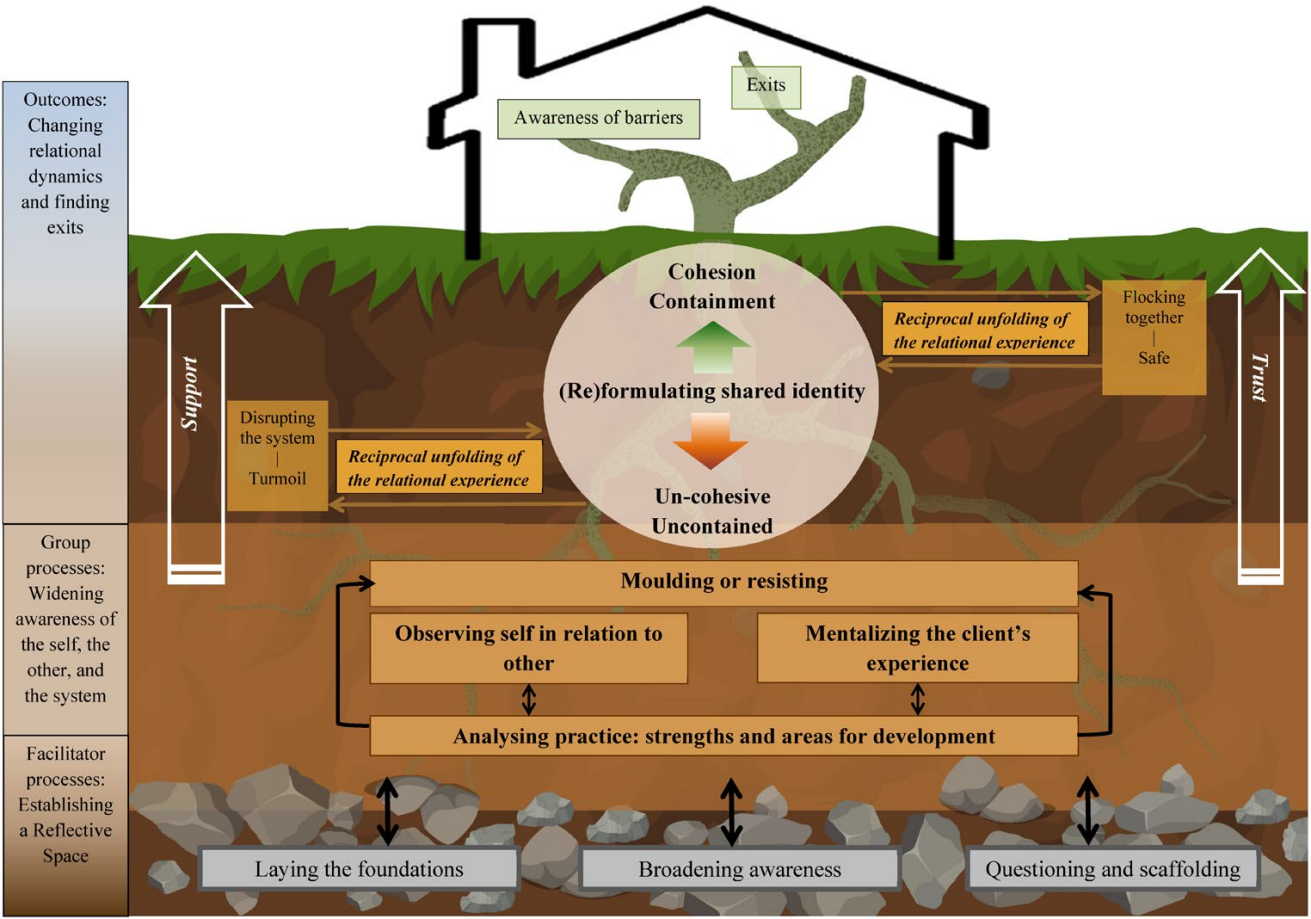
Constructed model of cognitive analytic RP.

Within the initial coding of the qualitative data, 245 codes were generated. Through focused and theoretical coding, 11 subcategories were constructed and condensed to form three main categories (see Figure 2 for the final model). The CARP and focus group data have been combined with illustrative quotes from both.

#### Facilitator processes: establishing the reflective space

This category had three sub‐categories, representing the array of facilitator behaviours encouraging staff to take up an observational stance in their care work. These processes were non‐linear, as they were used interchangeably across each session.

### Laying the foundations

The facilitator set the scene, through articulating the purpose of the CARP session, explaining confidentiality, and opening the floor for participants to bring topics. Participants were invited to ‘check‐in’ to share feelings and perspectives on the status of the team.

[RP] F So reflective practice is just about reflecting on your practice, so it can be anything, a leadership thing, it could be about the young people, it could be about your specific teams, so you can sort of use it for any way you want really; it's an open invitation. I don't have a strict sort of agenda. Erm, so I don't know if you want to start doing a quick sort of check in? Where you're at and how things are going? Then we can sort of see where it moves.

This process often led to participants identifying areas to focus on within the session, which was named by the facilitator.

[RP] F Yeah, so is it worth having a think about [YP] and what are some, some of the inconsistencies are?

From this, the facilitator invited participants to confirm whether identified topics would be a valuable focus, enabling opportunity for further clarification. An important foundation was the expectation of honest communication, through the facilitator establishing a confidential space, without external disruption or intrusion.

### Broadening awareness

This was the process whereby the facilitator introduced ideas or viewpoints not already in discussion and most often involved drawing participants' attention to care‐related emotional and intrapersonal processes.

[FG] P11 I think [the facilitator] touches upon it more than we do, because they talk about the impact that this job does have on us personally, and because we do it every day, we don't necessarily, it becomes normal…your first week of working here, you're like, “what is going on?”, but like, this is just normal now, so I think [the facilitator] helps us, reminds us, that it does affect your personal life

This also included the facilitator attempting to introduce the voices of YPs not present within the session, usually by inviting participants to consider the perspective of that individual.

[RP] F And in terms of the kids, what are they like in terms, ‘cos they, you know, they'll all have their views on this team, this team, this team, you know… do you get a sense, in terms of what the kids feel like within your team?

This process encouraged participants to broaden their narrative, through exploring relational dynamics from different perspectives; see ‘mentalising the experiences of YP's’ subcategory.

### Summarising and scaffolding

Within this subcategory, the facilitator shared CAT theory to conceptualise group discussions and extend ideas.

[RP] F That's really containing isn't it, in terms of how that makes you feel, if you've got, you know, think of the CAT training we did and the reciprocal roles, do you remember when we did that training about how the other feels in relation to you? If you as a leader are containing, everyone else is going to feel contained.

For example, through using the ‘boundary seesaw’, the facilitator provided a theoretical scaffold for participants to reflect on their relational approach with the YP.

[RP] F Do you remember this [boundary seesaw]? So, the idea is, this is just a really helpful model for thinking about where you are, sort of within yourselves, but also as a team.

After inviting these reflections, the facilitator encouraged participants to think about how they could use these reflections to inform their practice.

[RP] F The aim is that each of you can stay in this green zone, of being able to offer sort of, nurture and care, whilst at the same time being able to put in the boundaries, ‘cause every young person, as you know, they need the boundaries, but they also need the nurturing care alongside it.

This scaffolding towards relational awareness permitted participants to share perspectives on each other's relational style and consider the relational functioning of the team. Through this, participants engaged in ‘group processes: widening awareness of the self, other and system’.

#### Group processes: widening awareness of the self, other and system

This category had four sub‐categories, representing the array of mechanisms, topics and approaches demonstrated as part of the reflective process. These were often interlinked, rather than isolated, resulting in a layering of reflection using different perspectives.

### Analysing practice: reflecting on strengths and areas for development

Participants shared ideas around practices perceived as a relative strength or needing further development, at both the individual and team level.

[RP] p11 Yeah I feel like, this is across teams, and we're guilty of this as well, I feel like the enrichments are not very meaningful, I feel like sometimes it's just to fill time, “we'll just do this”, like, enrichments to me, I think should probably be a bit more meaningful, because ultimately these should be teaching children to build life skills.

Analysing practice also involved reinforcing what participants perceived to be good practice, which provided an opportunity to recognise these skills as resources and to enable change elsewhere.

[RP] P1 I will say something, I think [P4]'s done really well building those relationships up with some of the kids

F Well that's a fantastic start, and I think that is the biggest key isn't it, it's money in the jar, you get the rewards

P4 That's one of the best things [P1]'s ever said to me, “make sure you've got them relationships, 'cause without that you've got nothing”

Building good relationships within the team and with YPs, was frequently described as an integral relational resource creating a foundation for change. Participants engaged in an active process of sharing similar and/or different perspectives, to establish the direction and content of ‘good practice’.

[RP] P13 I mean a good shift for me, people would say “we haven't had a restraint”, nah, that's bollocks, that's not a good shift for me, a good shift for me is you get the young people, the young people are going to bed safe, they've got a smile on their face

### Observing the self through the other

Within this process, participants were developing knowledge about the ‘self’ (i.e., an individual or team) through team‐to‐team or peer‐to‐peer comparisons. Through recognising differences between the ‘self’ and the other, participants identified opportunities for change.

[RP] P5 I think with myteam and with [participant's name] team, we're both very, very different and I think we both need to try and come to some middle ground, so my team, are probably, well, they're definitely not as good as your team, at sticking to their times, making sure everything gets done, your, your, enrichments and everything are really rigid and my team is not there yet, but from some comments from some people on your team, our team get out on time, like, we're more efficient with the paperwork, and I just think we need to kinda, take a bit from each other's teams, to get a middle ground

This was often facilitated by the ‘summarising and scaffolding’ process, where invitations to use CAT concepts allowed participants to recognise differences in how they were perceived by others.

[RP] P8 I put myself in a different place to where others put me [on the boundary seesaw], and I think it opened my mind a little bit more, to my own factors and stuff.

This process interlinked with the ‘moulding or resisting’ and ‘(re)formulating a shared identity’ subcategories, as participants recognised their individual differences, whilst also seeking to establish team cohesion.

### Mentalising the experience of YPs

Participants attempted to understand their experiences of the YP and understand relational dynamics between the YP and staff. This process was encouraged by the facilitator (see ‘broadening awareness’ subcategory), through inviting reflections on how the YP might experience the team.

[RP] F What do you think the impact is in terms of the message the young people get from that?

P5 Er, well

P2 I'll be honest, and I'll say it, they think you're soft

Within this category, participants also attempted to disentangle contextual and longitudinal influences on YPs presentations. Through analysing ‘here‐and‐now’ contextual dynamics, participants considered the function of certain behaviours.

[RP] P1 And it's like [YPs] not here, his eyes become dilated, he's just gone

F Gone, yeah

P1 And it's like he gets, I don't know how to describe it, it's almost like he wants the restraint, and now we're not doing that, that's not happening

F Yeah

P1 But sometimes it's almost like he's pushing for that containment

Participants transferred their understanding from one YP to another. This demonstrated a legacy of how YP shaped staff's attempts to understand their experiences and highlighted the reciprocal learning that could occur between staff and YPs.

#### Moulding or resisting

Participants described pressures to develop in line with the wider team and fit the mould laid out by the secure system. Participants often used mechanical language, describing themselves as a ‘small cog’ in the ‘larger cog’ of the service. Participants spoke about entering the unfamiliar context of the care system, which presented a dilemma of whether staff would mould to, or resist, the system.

[FG] P17 When I first started I said “oh my god, everything's military and I can't cope with it” but like, now I've adapted and I see how, like, because it is military, how well we work, you can see why it works for the kids when you set up your shift, the kids know exactly what's going off when you as a staff member know what's going off

The consequences of staff members within the service going ‘off tangent’, rather than moulding to expectations, was seen as detrimental because it caused system disruption; a ‘chain reaction’ impacting each ‘cog’ in turn.

[FG] P18 If I go off tangent from these, then I'm not a team player

P16 Mmm, if people don't do what they're supposed to do and they go off and do their own thing it has an effect.

P17 A chain, chain, you know what I mean chain reaction, so to speak.

For team members seen to be resisting the mould, participants felt that these individuals should adapt to the team's way of working to keep the ‘well‐oiled machine’ working.

[RP] P1 They need to adapt to our way of working a bit more

F Okay so, there's a bit of a‐

P1 ‘Cause we have cogs in a team, it's a bit like a watch, if one of the cogs isn't‐

F Erm, okay, and is that then, does that have an impact?

P1 Mmm, it has a massive impact

F ‘Cause like you say it's like a cog isn't it, you have your little cog and then there's all the bigger cogs, and then you've got the bigger cog of [the SCH]

P1 Mmm…If people don't do what they're supposed to do and they go off and do their own thing, it has an effect

The content of this subcategory can be seen as existing in relation to the ‘(re)formulating a shared identity’ subcategory, as resisting the mould was experienced as challenging the cohesion sought by the team.

#### Outcomes: changing relational dynamics and finding exits

This category had four sub‐categories, reflecting the primary outcomes of RP and included participants reflecting on how their team dynamics influenced relational processes with YPs and intentions to alter practice based on the content of RP.

### (Re)formulating group identity

A function of CARP sessions seen as integral by participants was to ‘(re)formulate a shared identity’. Participants explored their sense of team identity and discussed how periods of change caused disruption to this process, which could leave participants feeling uncontained.

[RP] P7 Erm… it's same with anything ain't it, when you're a team, just a team in anything, sport, work, whatever it is, you, you, you find yourself a niche don't you? A spot, something that you do, that you feel…

F Where your team are in that stuff?

P7 Yeah, so it's hard to create that when you're all over the place

Within this category, participants named RP as an opportunity to facilitate togetherness, through sharing experiences and identifying ways to collectively develop.

[FG] P13 It [reflective practice] gives you a togetherness, you're in a like mind‐set going out, and everybody has got your back

Participants revisited the process of ‘mentalising the experience of YPs’ to consider why this was important, beyond their own sense of feeling supported. Within these reflections, the context of the secure environment influenced the sense of needing to be a ‘strong unit’.

[FG] P3 You just feel together, you portray that, the kids see you coming on, you're a unit and you're together

P11 Strong

P3 I know it sounds silly, but if like, if you feel like you're going to war, like, you're not going to battle on your own

P11 Kids would like to divide and conquer, but if you're a strong unit and a strong team, that's not happening

The importance of establishing a ‘strong’ and cohesive identity appeared to sit in relation to the care environment in which threats to safety were anticipated.

### Reciprocal unfolding of the relational experience

Participants reflected on how relational dynamics of the team had reciprocal influence on the YPs experience of care. This subcategory interconnected with ‘(re)formulating a shared identity’ in that team cohesion operated as the ‘glue’ for harmonious staff relationships, consequently creating a more therapeutic home.

[FG] P18 I think people forget, this is the children's home, but as a professional, some might say it, some might not, but this is my home, these are my peers that I'm working with, I'm living with my colleagues, so we're all, it's all harmonious. For me, to have that team identity, it's leading to us being cohesive, if we're cohesive like a glue, those kids get the best

P16 It's like a marriage isn't it

P18 Yeah

P16 ‘Cause we're all looking after the young people. If the marriage is bad, the home life is bad isn't it. That's how I see it.

The language used to describe relationships was often reflective of a ‘home’ environment, with staff being ‘parents’ to the YPs and with due appreciation of the importance of these relationships.

[RP] P1 I am noticing when we have [CARP], that staff and young people know where we stand, ‘cause when I'm working across other teams, the staff haven't got a clue what shift set up or structure is, and that's what sends the kids into turmoil

When the team was not cohesive there was recognition that this could leave YPs in ‘turmoil’ and when there was consistency, YP felt typically ‘safe’.

[RP] P2 ‘Cause the [YPs] know where they stand, they know what they're doing, they know the routine, and because as a team we are so consistent, they know what they can get away with and what they can't

F So what does that make the young people feel?

P2 It makes ‘em feel safe.

These reflective processes were linked with the ‘mentalising the experience of YPs’ subcategory but extended to recognise relational experiences as being both reciprocal and operating in parallel.

### Building trust and feeling supported

Participants perceived trust and support between team members as integral to establishing and maintaining cohesion and a shared identity.

[FG] P18 Once you've got that trust in your team

P9 You've got the same aims

P18 They are the ingredients to make cohesion, they're the vital ingredients, if you don't have them vital ingredients there, you don't have cohesion in us as a team, they're the vital ingredients

Participants highlighted how the context of the care environment strongly influenced the importance of the team feeling able to trust and support each other. In part, the function of RP was to offer a facilitated space for honest conversations to build trust.

[FG] P13 ‘Cause when I go through there, I'm trusting you to support me, cause anything could happen, somebody could get stabbed, somebody could get kicked, somebody could punched, and it's happened…you have to support each other and have that trust within the team, that I trust you 100% that you'll have my back, that's with anything, if I'm in a restraint, somebodies got to take over, you know? And that comes down as a team, and as an individual member of staff, we need to have that honest conversation and this [CARP] is the forum for it

### Establishing exits and overcoming barriers

Participants negotiated establishing care solutions as ‘exits’ to the issues discussed. Within this process, it was often vocalised that change was unsustainable or outside of staff control.

[RP] P11 It doesn't matter what we do here, as soon as [YP] goes out, they'll just rule [their parents] again

To mitigate against this inertia, the facilitator would often attempt to revisit the ‘broadening awareness’ process, to introduce ideas that could shift perspectives.

[RP] F When you take all this away, has this kid changed and will it all be hunky dory? No, absolutely not. But what you have given them is an experience of some consistency and some containment and building some relationships, you know, they'll look back at [the SCH] as one of the best times in their life, I genuinely think that… [YP]'s said similar

Many identified exits were accompanied by recognition that team leaders would need to scaffold the development of junior members of the team.

[RP] P5 I know the areas of development for myself and my team, I know what they need to do better and I know what I need to do to get them there, but I just think, [participant name] is my big example, I can, I can, model to show them, I can watch them do it the next time, they see it happen differently, and it all unravels and goes back to where they were before

Establishing change also required consistency of modelling across teams to cement new ways of working into practice. From this, a further function of cohesion was to facilitate change as part of a system‐wide process, rather than an isolated endeavour. This demonstrated how practice‐based changes were seen as consistently underpinned by a relational and reciprocal processes between components of the care system.

#### Helpfulness and cohesion measures

**FIGURE 3:**
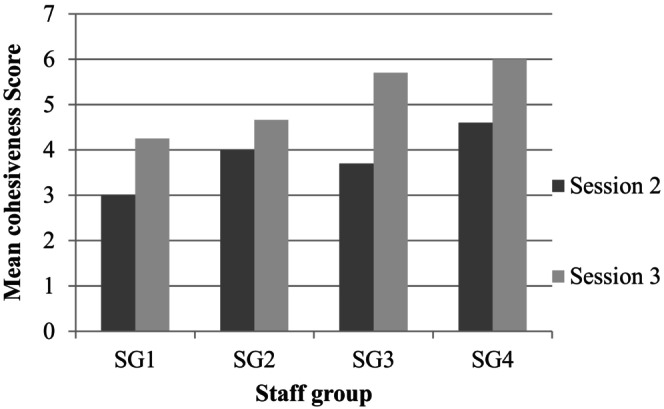
Mean cohesiveness scores for each CARP sessions across participants.

To assess whether the constructed categories were aligned to participants' perceptions of the helpful/unhelpful elements of CARP and team cohesiveness, the GEM‐RP and qualitative HAT responses were coded and, where appropriate, aligned to the constructed categories. A total of 28 HAT responses were obtained. These mostly concerned ‘helpful’ or ‘important’ events, with a single hindering event (i.e., a ‘high alarm’ causing disruption). The mean HAT score across all CARP groups was 8.31 (*SD* = 0.76) and a Kruskal–Wallis one‐way ANOVA found no significant between group differences, *H*(7) = 10.75, *p* = .15. No new codes arose from the analysis of qualitative HAT data indicating saturation. Sessional mean GEM‐RP scores are reported in Table 2. Cohesiveness (see Figure 3) increased from session 2 (*M* = 3.8), to session 3 (*M* = 5.1). The pairwise analysis of sessions in the Kruskal–Wallis one‐way ANOVA indicated these changes were statistically significant, *H*(1) = 4.18, *p* = .04. The GEM‐RP results provide additional confirmation of the constructed GT model. In particular, the increase in group cohesion appears to corroborate the ‘(re)formulating a shared identify’ as an integral RP process and outcome for staff.

**TABLE 2:** GEM‐RP mean team cohesion scores for each staff group.

Staff group	Number of responses	Session 2: mean GEM‐RP score	Number of responses	Session 3: mean GEM‐RP score
1	2	3	4	4.25
2	3	4	3	4.66
3	4	3.7	4	5.7
4	3	4.6	3	6
		*M* = 3.8 (*SD* = 0.67)		*M* = 5.15 (*SD* = 0.83)

## DISCUSSION

The current research sought to produce a GT model of CARP groups to better understand whether, and how, this approach facilitates reflective and relational awareness in staff caring for vulnerable YP. Using a bottom‐up and data‐driven approach, the direct analysis of session content found that establishing team cohesion was key outcome. The GT model produced therefore contributes to the growing literature of CAT as a helpful indirect organisational intervention, particularly through the CAT approach enabling increased cohesion within staff teams (Caruso et al., 2013; Doyle et al., 2019; Kellett et al., 2014; Thompson et al., 2008). The constructed model proposed both linear and reciprocal processes occuring between facilitator and participants. The constructed main categories illustrated that through ‘establishing a reflective space’, staff were able to engage in processes that widened their ‘awareness of the self, other, and system’, to ‘change relational dynamics and establish exits.’

A distinguishing feature, and therefore significant contribution of CAT, is that it is a relational model of mental health (Ryle & Low, 1993) and in the current study the CAT model was intended to structure RP groups (Dallos & Stedmon, 2002). The key issue is therefore the degree to which CARP differs in practice from generic RP. The fidelity check evidenced that the CARP groups contained a sufficient degree of CAT to confidently ascertain that the sessions were not generic RP. This is also evidenced in the finding that two out of the three main categories were relational in nature (i.e., awareness of systems and enabling change to relational dynamics). It is worth noting that the CCAT (Bennett & Parry, 2004) has 10 domains and only one of these is model specific. Therefore, in the context of CARP, some of the discussions and processes would be akin to generic RP and some will be model specific. It was not possible to apply the extant CCAT scoring system in the current project because of the alterations made to the measure.

The three interrelated change processes facilitated by CARP are perhaps best understood in terms of the zone of proximal development (ZPD), which is used to pace and organise CAT interventions (Ryle & Kerr, 2020; Vygotsky, 1978). It is acknowledged that the training intervention regarding CAT principles was only a single day, and more input might have enabled staff to more easily and rapidly engage with the CARP. The team's ZPD for care represents the difference between what the team would be able to deliver independently, compared to their potential care ability when support and scaffolding are available from consultation, such as RP. The three GT categories could therefore be seen as the participants and facilitator engaging in reciprocal processes to scaffold their understanding via experiential learning to widen their ZPD for care. These elements appeared to provide integral supporting pillars for the scaffolding process, allowing participants to feel supported to work outside of their ‘comfort zone’, but not become ‘overwhelmed’. The interrelated processes identified within the GT model demarcates key differences with other models of RP, through highlighting analysis of the more relational and reciprocal processes of care. When CAT practitioners go from delivering one‐to‐one therapy to more group based and consultative approaches, then there are personal ZPD issues that need to be considered and supported through relevant supervision.

In terms of study limitations, the use of focus groups may have placed restrictions on the data through limiting the narratives shared. Focus groups can lead to more normative discourses, during which conflicting or contentious positions are unlikely to be expressed (Smithson, 2000) or discourses being more driven by dominant voices (Smithson, 2000). The current study was set in a single SCH in England, and this may limit the transferability and generalisability of findings. There could have been wider fidelity sampling, and the quantitative measures could have benefited from a measure of individual team member well‐being. It is acknowledged that cautious interpretation of CAT word frequency results reported is required when assessing adherence to CAT, as this is a crude measure, and that CAT theory can be used without necessarily using CAT terminology. The fact that a grounded theory of the qualitative data was performed means that the project was a formal piece of research and not a service evaluation, in which simple thematic analysis would have sufficed. Future research should (a) seek to explore whether YPs experience any changes in care practices following RP and whether this influences outcomes, (b) generate larger SCH samples, (c) have a control SCH to compare outcomes against, (d) use individual interviews as opposed to focus groups (e) compare processes and outcomes in RP groups using different theories, (f) develop and evaluate a competency model and associated measure of CARP and (g) use the presented GT to guide semi‐structured interviews with staff attending CARP within other SCH settings. Future studies need to emphasise identifying the model specific aspects of CARP.

To conclude, this research has met its aim of providing a theoretical model for CAT‐informed RP. The constructed GT model highlights the reciprocal and relational focus of CARP , which is used to understand and then change the dynamics of caring for YP with trauma and associated emotional difficulties. The approach appears to enhance team cohesion and widen relational awareness of the reciprocal processes occurring between staff, YPs and wider organisational systems. There is clearly a need for future more controlled research that seeks to evaluate and then maximise how RP can be best used to inform the care that is delivered to YP in a CYPSE context. .

## AUTHOR CONTRIBUTIONS

**Sasha Priddy:** Conceptualization; investigation; writing – original draft; methodology; formal analysis; project administration. **Stephen Kellett:** Conceptualization; investigation; writing – review and editing; methodology; supervision. **Shona Goodall:** Conceptualization; investigation; supervision. **Rachael Cotton:** Project administration; investigation.

## CONFLICT OF INTEREST STATEMENT

None of the above authors has any conflicts of interest to report regarding this piece of work.

## Supporting information


Appendix S1.

## Data Availability

The data that support the findings of this study are available on request from the corresponding author. The data are not publicly available due to privacy or ethical restrictions.
