# Supplementary material for: A grounded theory of cognitive analytic reflective practice groups
Source: Psychol Psychother. 2024 Nov 11;98(1):40–57. doi: 10.1111/papt.12557 (PMC11823393; doi:10.1111/papt.12557)
Supplement: Supplementary file 1 — Appendix S1. [file PAPT-98-40-s001.zip › Supplementary Material Table One.docx]

Supplementary Material Table One; *CARP adherence descriptions and ratings*

| **CCAT Domain** | **Relevance for CARP** | **Carp 4**  (Yes -present/observed) | **CARP 6**  (Yes -present/observed) |
| --- | --- | --- | --- |
| Phase specific therapeutic tasks | A potential area of focus is identified with group members without premature focusing or imposition of the facilitator’s own agenda | Yes | Yes |
| Theory practice links | The facilitator is noticing and taking opportunities to use the CAT model to structure sessions and conceptualise CARP group member’s experiences. Simply, is CAT guiding this work?  The facilitator uses CAT theory to conceptualise the dynamics in the facilitator/group relationship | Yes  Not observed | Yes  Not observed |
| CAT specific tools and techniques | The facilitator encourages CARP attendees understanding and application of relevant CAT tools and techniques to aid reflective processes (i.e. the boundary seesaw, use of reciprocal roles to guide interactions) | Yes | Yes |
| External framework | The facilitator effectively establishes and maintains a containing frame for CARP sessions and observes boundaries within the professional relationship.  Are there parallel processes in the facilitator/group relationship that mirror the therapeutic relationship with the client in terms of boundary violation or enmeshment? If present, these appear to be recognised and worked with. | Yes  Not observed | Yes  Not observed |
| Common factors | The facilitator establishes and maintains relationships with CARP attendees that include warmth, supportiveness, and attentiveness to the challenges of the work and continued personal development | Yes | Yes |
| Respect, collaboration and mutuality | Mutuality is evident in the CARP group, in relation to how the facilitator shares and develops the use of CAT tools.  Reflecting together in the CARP session feels like an authentic, joint learning encounter and reflects a genuine shared purpose and sense of teamwork | Yes  Yes | Yes  Yes |
| Assimilation of warded off emotions and problematic states | The CARP facilitator encourages, contains, conceptualises and assimilates the CARP attendees experience of their work and the reflective practice process | Yes | Yes |
| Identifying and managing threats to the therapeutic alliance | The facilitator uses the model so that they are alive to identifying, and then exploring reciprocal role enactments within CARP sessions, that represent threats to the CARP attendee’s learning/development, pose a threat to the facilitator/group alliance or are an important opportunity for developing awareness. | Not observed | Not observed |
| Making links and hypotheses | The facilitator enables CARP attendees to see their relational ZPD both within and across their therapeutic relationships, so that they can become more aware of unhelpful procedures with clients and peers.  The facilitator helps CARP attendees to see any parallel processes between the position they find themselves in with the client, their peers and the position they find themselves in during CARP sessions | Yes  Not observed | Not observed  Not observed |
| Awareness and management of own reactions/emotions | The CARP facilitator and CARP attendees reflect on and manage their emotional state during CARP sessions and use this information to inform the process of reflective practice | Yes | Not observed |
