# Supplementary material for: A grounded theory of cognitive analytic reflective practice groups
Source: Psychol Psychother. 2024 Nov 11;98(1):40–57. doi: 10.1111/papt.12557 (PMC11823393; doi:10.1111/papt.12557)
Supplement: Supplementary file 1 — Appendix S1. [file PAPT-98-40-s001.zip › Supplementary Material Table Three.docx]

Supplementary Material Table Three; *examples of memo entry and associated transcript extracts*

| **CARP Session** | **Transcript extract** | **Associated memo** |
| --- | --- | --- |
| Reflective practice 5 | p7: Yeah, but it’s probably always going to be ‘ent it, so then you just have to take it on yourself, and that, that, this is my p8: Take on it p7: Take on it, so then if you come somewhere else and it don’t, and maybe that’s not there, just don’t even ask the fucking question, just like, "no you can’t and it’s as simple as that", and then if f: You mean don’t, so say if [participant] is on [participants] team and a young person asks for orange juice and you just say "no, that’s, that’s not a thing", rather than going to the team leader to ask | Participants discussing how they will not mould to fit with other teams when they work across the service. This seems to have interesting implications for cohesion and demonstrates where the cohesion is most desired (i.e. within-team, rather than between-team) |
| Reflective practice 3 | f: A bit of both, yeah, and have you noticed that P7, in terms of P8? p7: Yes, yeah, I think it’s pretty natural thing, like, erm, when we’ve had a big change over in terms of kids and stuff, its and opportunity ‘ent it, to bring yourself back to where you f: Want to be p7: Want to be f: Yeah | Young people also seem to be part of the pulls to mould or resist. Changes in the cohort of young people offer an opportunity to reshape oneself and return to “where you want to be |
| Reflective practice 3 | p2: Yeah, he looks after his staff, they look after the young people f: Yeah yeah p2: He’s consistent and he means what he says and he says what he means | Interesting illustration of reciprocal roles, seems to speak to the idea that the relationship between team leader and the staff team is ‘internalised’ by staff, so that it can then be enacted with the young people (other-to-self, then, self-to-other). |
