# Supplementary material for: A grounded theory of cognitive analytic reflective practice groups
Source: Psychol Psychother. 2024 Nov 11;98(1):40–57. doi: 10.1111/papt.12557 (PMC11823393; doi:10.1111/papt.12557)
Supplement: Supplementary file 1 — Appendix S1. [file PAPT-98-40-s001.zip › Supplementary Table Four.docx]

Supplementary Table Four: *example of a reflective dairy entry*

| Date and time | I visited the SCH today in order to begin transcribing. Noticed that the experience of entering the SCH environment seemed to begin to alter and influence my expectations of how the context might influence CARP, so felt it was important to document this. I was struck by the amount of secure restrictions, with each door having to be mechanically locked and unlocked, which makes moving anywhere a long process and also continuously reminded me of the context that I was in; (i.e. this level of ‘safety’ is required due to the level of ‘risk’. I thought about how this process of movement being restricted might impact on how individuals within the context experience their own sense of autonomy, for staff and YP’s, both physically, cognitively and emotionally. I also thought about how people might feel relationally much more distant, when there are so many locked barriers between people within the SCH. |
| --- | --- |
