# Supplementary material for: A grounded theory of cognitive analytic reflective practice groups
Source: Psychol Psychother. 2024 Nov 11;98(1):40–57. doi: 10.1111/papt.12557 (PMC11823393; doi:10.1111/papt.12557)
Supplement: Supplementary file 1 — Appendix S1. [file PAPT-98-40-s001.zip › Supplementary Table Two.docx]

Supplementary Table Two; *semi structured interview schedule*

| **Main areas** | **Questions and further prompts** |  |
| --- | --- | --- |
| Experience of CARP | Can you tell me a little bit about your experience of engaging in the reflective practice group (RPG) at [the SCH]?  • Prompt: how would you summarize the overall experience? What does reflective practice mean to you? Has anyone engaged in reflective practice before?  • Follow up: Did you notice any differences between your previous experience and the reflective practice groups at [the SCH]?  • Follow up: What impact do you think these differences had? What, if anything, did you value about engaging in the reflective practice groups? Can you tell me about what factors, if any, influenced your ability to use the group space to reflect? |  |
| Personal and professional development | How, if at all, do you think that taking part in the RPG influenced your professional development?  • Prompt: in relation to your understanding of the job or your practice?  • Follow up: What happened within the RPG to enable this development?  • Follow up: Did you experience anything that inhibited your professional development? How, if at all, do you think that taking part in the RPG influenced your personal development? Prompt: in relation to understanding yourself, your emotional reactions, managing your wellbeing?  • Follow up: What happened within the RPG to enable this development?  • Follow up: Did you experience anything that interfered or inhibited your professional development |  |
| Impact of CARP on practice | In what ways, if any, has CARP influenced the ways in which you engage with your work within the secure children’s home?  • Follow up: Can you describe anything that happened within the RPGs, which facilitated these changes? |  |
| Identity | What has it been like to come together to share the RP space? How, if at all, do you think that taking part in the RPG influenced your understanding of your role within the team? How, if at all, do you think that taking part in the RPG influenced your identity as a team?  • Follow up: What, if any, ideas or tools did the facilitator bring that influenced this |  |
| Wider context | How, if at all, do you think engaging in the groups influenced your awareness of relational dynamics within the SCH? How, if at all, do you think engaging in the groups influenced your awareness of how the SCH ‘system’ influences your practice? |  |
| Ending questions | In what ways, if any, has the COVID-19 pandemic influenced your experience of taking part in the RPG? Is there anything else you feel that I should know in order to understand your experience of engaging in CARP sessions whilst working within a secure children’s home |  |
